# Genome-wide identification of MADS-box gene family in sacred lotus (*Nelumbo nucifera*) identifies a *SEPALLATA homolog* gene involved in floral development

**DOI:** 10.1186/s12870-020-02712-w

**Published:** 2020-10-29

**Authors:** Zhongyuan Lin, Dingding Cao, Rebecca Njeri Damaris, Pingfang Yang

**Affiliations:** 1 State Key Laboratory of Biocatalysis and Enzyme Engineering, School of Life Sciences, Hubei University, Wuhan, 430062 China; 2 Institute of Oceanography, Minjiang University, Fuzhou, 350108 China

**Keywords:** Floral organogenesis, Genome-wide analysis, Lotus, MADS-box, *SEP3*

## Abstract

**Background:**

Sacred lotus (*Nelumbo nucifera*) is a vital perennial aquatic ornamental plant. Its flower shape determines the horticultural and ornamental values. However, the mechanisms underlying lotus flower development are still elusive. MADS-box transcription factors are crucial in various features of plant development, especially in floral organogenesis and specification. It is still unknown how the MADS-box transcription factors regulate the floral organogenesis in lotus.

**Results:**

To obtain a comprehensive insight into the functions of MADS-box genes in sacred lotus flower development, we systematically characterized members of this gene family based on the available genome information. A total of 44 MADS-box genes were identified, of which 16 type I and 28 type II genes were categorized based on the phylogenetic analysis. Furthermore, the structure of MADS-box genes and their expressional patterns were also systematically analyzed. Additionally, subcellular localization analysis showed that they are mainly localized in the nucleus, of which a *SEPALLATA3* (*SEP3*) homolog *NnMADS14* was proven to be involved in the floral organogenesis.

**Conclusion:**

These results provide some fundamental information about the MADS-box gene family and their functions, which might be helpful in not only understanding the mechanisms of floral organogenesis but also breeding of high ornamental value cultivars in lotus.

**Supplementary Information:**

The online version contains supplementary material available at 10.1186/s12870-020-02712-w.

## Background

*Ne****l****umbonaceae* is one of the smallest families in flowering plants, which consists of only two species named as *Nelumbo nucifera* Gaertn. and *Nelumbo lutea* Pers, respectively [1, 2]. *N. nucifera* is also called sacred lotus in Asia based on its significance in Buddhism and Hinduism [3]. Besides, lotus is also an important horticultural plant with ornamental, nutritional, and medicinal values [3]. In reality, there are three major categories of lotus, namely flower, seed, and rhizome lotus. Flower lotus is a very important aquatic ornamental plant in Southeast Asia because of its variable flower color and shape, which is among the top ten famous flowers in China. Flower shape is largely determined by the arrangement of its four basic constitutive organs, sepals, petals, stamens, and carpels. The petal number, size, and shape contribute greatly to the flower shape. In lotus, there are many transition petal shapes. Based on lotus flower morphology, there are four groups, named as few-petalled, double-petalled, duplicate-petalled, and all-double-petalled, respectively [1, 2]. Petaloid is one of the key features that are selected in the breeding of ornamental plants, based on which numerous lotus cultivars showing petaloid differentiation, such as stamen petaloid and carpel petaloid have been obtained. This petal shape transition feature makes lotus an ideal plant for studying floral development, especially floral organogenesis.

Since the identification of the first key transcription factors (TFs) in floral organogenesis [4], a series of TFs controlling floral organ specification have been characterized, which have resulted in the well-known classic ‘ABC/DE’ model [5–11]. Different combination of A, B, C, and E classes genes determines the floral organ specification, such as sepals, petal, stamen, carpel, and ovule controlled by A + E, A + B + E, B + C + E, C + E, and D + E, respectively [12–15]. A subset of functional genes in this model is comprised of class A (*APETALA1/FRUITFULL*, *AP1/FUL*), class B (*APETALA3/PISTILLATA*, *AP3/PI*), class C/D (*AGAMOUS/SEEDSTICK/SHATTERPROOF1/2*, *AG/STK/SHP1/2*), and class E (*SEP1/2/3/4*) [9, 13, 16].

Except for a putative A-class gene *APETALA2* (*AP2*), all the other known A, B, C, and E class genes encode MADS-box proteins [8, 17, 18]. The acronym MADS box stands for the initials of four loci, MINICHROMOSOME MAINTENANCE (MCM1) of yeast, AGAMOUS (AG) of *Arabidopsis thaliana*, DEFICIENS (DEF) of *Antirrhinum majus,* and SERUM RESPONSE FACTOR (SRF) of *Homo sapiens*, of which all members contain a conserved 58–60 amino acids M-domain region in the N-terminus [19]. In eukaryote, the MADS-box gene family is ubiquitous for development control [19]. Phylogeny divides the MADS-box gene family in two clades, type I and type II, which contain genes with SRF-like MADS domains or MYOCYTE ENHANCER FACTOR 2 like (MEF2-like) domains [20]. Type I MADS-box genes can be further classified into Mα, Mβ, and Mγ, whereas the type II MADS-box genes only exist in the plant kingdom [21] and are further categorized into MIKC^c^ and MIKC^*^ based on structure divergence of the intervening (I) region [20, 22]. In addition to MADS-domain (M), MIKC-type proteins generally contain another three common domain structures, including intervening (I) domain, keratin-like (K) domain, and C-terminal (C) domain, which together interact with other components and bind to CArG box to activate the expression of the downstream genes [15, 22–24].

Previous studies have shown the ubiquitous functions of MIKC^c^ genes in plant development [25–29]. Several MIKC^c^ genes, such as *AGAMOUS like 24* (*AGL24*) and *SHORT VEGETATIVE PHASE* (*SVP*), *FLOWERING LOCUS C* (*FLC*) and *MADS AFFECTING FLOWERING* (*MAF1/FLM*), *AGL15* and *AGL18*, and *SUPPRESSOR OF OVEREXPRESSION OF CONSTANS 1* (*SOC1*) were shown to control flowering time in *Arabidopsis* [30–34]. *TRANSPARENT TESTA16* (*TT16*) gene is involved in the pigmentation of the seed coat and embryo development [35]. *AGL6* genes play numerous roles in floral meristem regulation, floral organ, seed development, and male and female floral organ development [36]. Some of them also regulate the vegetative growth, such as *AGL12* is important in root development as well as in flowering transition [37], and *AGL17*-clade homolog genes regulate lateral root development [28, 38].

Because of their important roles in plant development, especially in floral organogenesis, characterizing the functions of different MADS-box genes has been one of the hotspots in plant biology community. To achieve this, systematic genome-wide analysis of MADS-box gene family is necessary, which have been widely conducted in many plants, such as Arabidopsis [22], rice [39], *Brassica rapa* [40], orchid [41], grapevine [42], and *Rosa chinensis* [11]. For lotus, the genome has recently been sequenced and released [43, 44], which facilitates further study on function characterization of gene or gene family. Similar studies have been conducted on the bHLH, R2R3 MYB, and GARS transcription factor families [45–47]. However, a genome-wide systematic analysis on the MADS-box gene family in lotus is absent, although few studies have been conducted focusing on some specific members of this gene family, including the *APETALA1-like* gene [48] and other floral organ identity MADS-box genes [49]. To obtain more comprehensive insight about the functions of MADS-box gene family in lotus development, especially in controlling its flower shape, we conducted a genome-wide identification of MADS-box genes, and systematically analyzed their distribution, phylogenetic relationship, gene structure, and expression profiles in the sequenced sacred lotus ‘China Antique’. Certain candidate MADS-box genes that might be involved in floral organ formation were selected for further analyses, of which a *SEP3* homolog was proven to be involved in the floral organ specification. These results might help to further understand the function of this gene family, especially in lotus flower development.

## Results

### Identification of MADS-box genes and their distribution in lotus genome

To obtain a general knowledge on MADS-box gene family in lotus, we first searched for them in the lotus genome database to determine the number of genes in this family [50]. A set of 52 candidates annotated as MADS-box genes (Table S1) were retrieved. Meanwhile, after bio-sequence analysis, the same number of putative MADS-box proteins was also derived. To accurately determine the members, after removing the candidates that aligned to the same sequence ID in NCBI (Table S1), a total of 44 *N. nucifera* MADS-box genes (*NnMADS1–44*) were confirmed by annotating them with *Arabidopsis* MADS-box gene names [22], of which some contained different transcript variants (Table S1). Some of them had segmental or tandem duplications (Table S2). Their non-synonymous (*Ka*) and synonymous (*Ks*) substitution rates were analyzed (Table S2) and the Ka/Ks ratio values were nearly close to one.

Lotus genome has been assembled into megascaffolds, including nine big and several small ones [43]. Among all the confirmed *NnMADS* genes, 36 were distributed in the top ten biggest megascaffolds 1–10 (Fig. S1a), and the remaining 8 genes were anchored in other small megascaffolds (Fig. S1b). Except for megascaffolds 3 and 7 that did not contain any *NnMADS* gene, megascaffold 2, one of the top largest megascaffolds (133.00 Mb) contained seven *NnMADS* genes; megascaffolds 1, 4, 5, and 6 all had 5 members (Fig. S1b). Similar to the distribution of R2R3 MYB [51], the density of MADS-box genes in the megascaffold is not uniform. Megascaffold 4 was the densest with 9.24 Mb/MADS-box gene while megascaffold 1 had the minimum density (51.00 Mb/gene) (Fig. S1c).

### Phylogenetic analysis of *N. nucifera* MADS-box genes

**Fig. 1:**
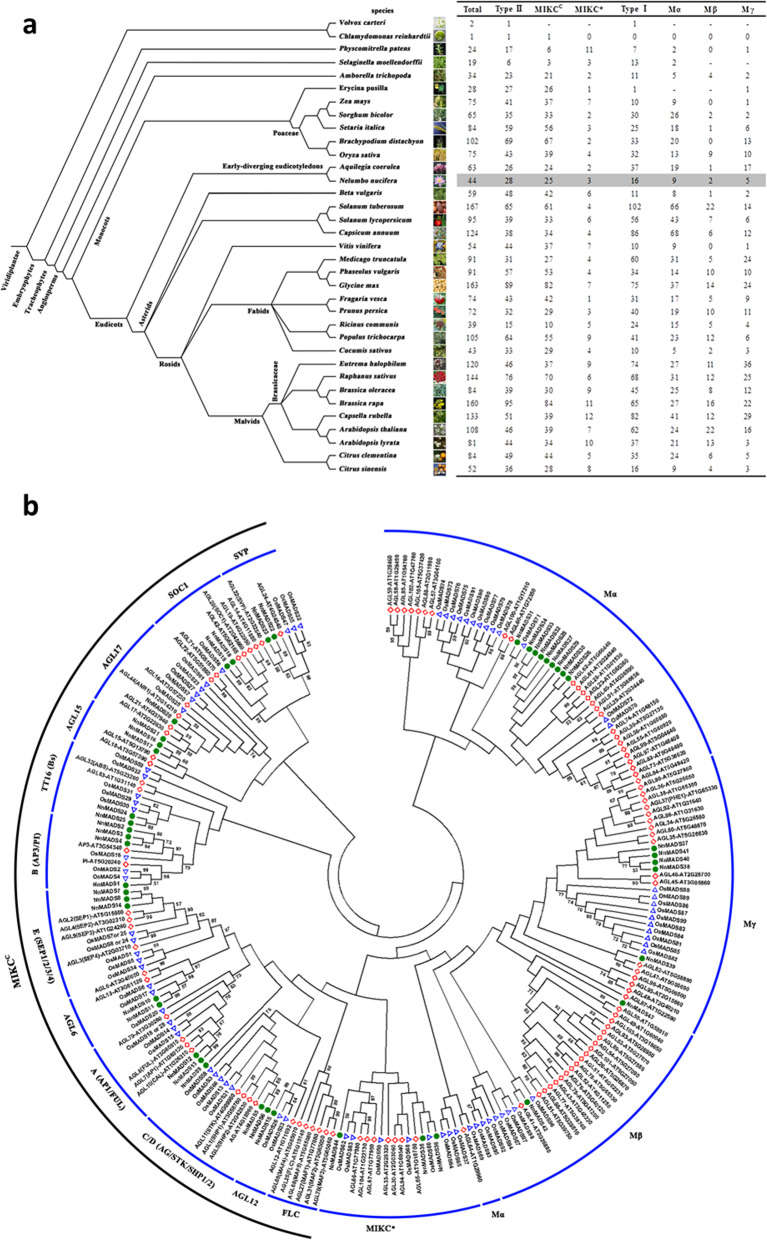
Classification and phylogenetic analyses of MADS-box family genes among different plant species. **a** The number of MADS-box family genes and their classification in 35 different plant species. The data for lotus, Arabidopsis, and rice are marked in green, red and blue, respectively, which were used for the following phylogenetic analysis. **b** Phylogenetic analysis of the MADS-box proteins from lotus, Arabidopsis and rice. The phylogenetic tree was constructed with the full-length protein sequences by the neighbor-joining method using MEGA software version 5.2. The digital number on each branch represents bootstrap scores for 1000 replicate analyses. A branch with a bootstrap score below 50 was considered unreliable and cut off. The text out of the blue and black lines represent different types or subtypes of MADS-box proteins

To-date, MADS-box genes in the genomes of 34 other plant species have been reported and summarized [40, 41, 52]. Among all the listed plant species, lotus contains a relatively small family of MADS-box genes (Fig. 1a), which is comparable with other basal species (Fig. 1a). To obtain a more comprehensive insight on this gene family in lotus, the full length of each MADS-box gene of lotus, *Arabidopsis,* and rice were downloaded from the NCBI database for further category and phylogenetic analyses. Based on the analysis of BLASTP against TAIR database, there were 16 type I (including nine Mα, two Mβ, and five Mγ) and 28 type II (including 25 MIKC^c^ and three MIKC*) MADS-box proteins in lotus (Fig. 1a and Table S1), and all the predicted MADS-box proteins contained different transcript variants belonging to MIKC^c^ (Table S1). Meanwhile, we also constructed the phylogenetic tree using sequences of all the predicted MADS-box proteins from the three species (Fig. 1b). The 25 MIKC^c^ type *NnMADS* genes (Fig. 1b) could be further grouped into twelve subfamilies: SOC1, AGL6, A (AP1/FUL), B (AP3/PI), C/D (AG/STK/SHP1/2), E (SEP), SVP, AGL12, AGL15, AGL17, TT6 (B sister), and FLC (Fig. 1b and Table S3). However, the FLC-like gene was absent in lotus (Fig. 1b). The subclade B included four members, both A and E contained three while the rest subclades contained two members except for AGL12 that had only one member (Fig. 1b and Table S3).

### Analysis of genes structure and conserved motifs

**Fig. 2:**
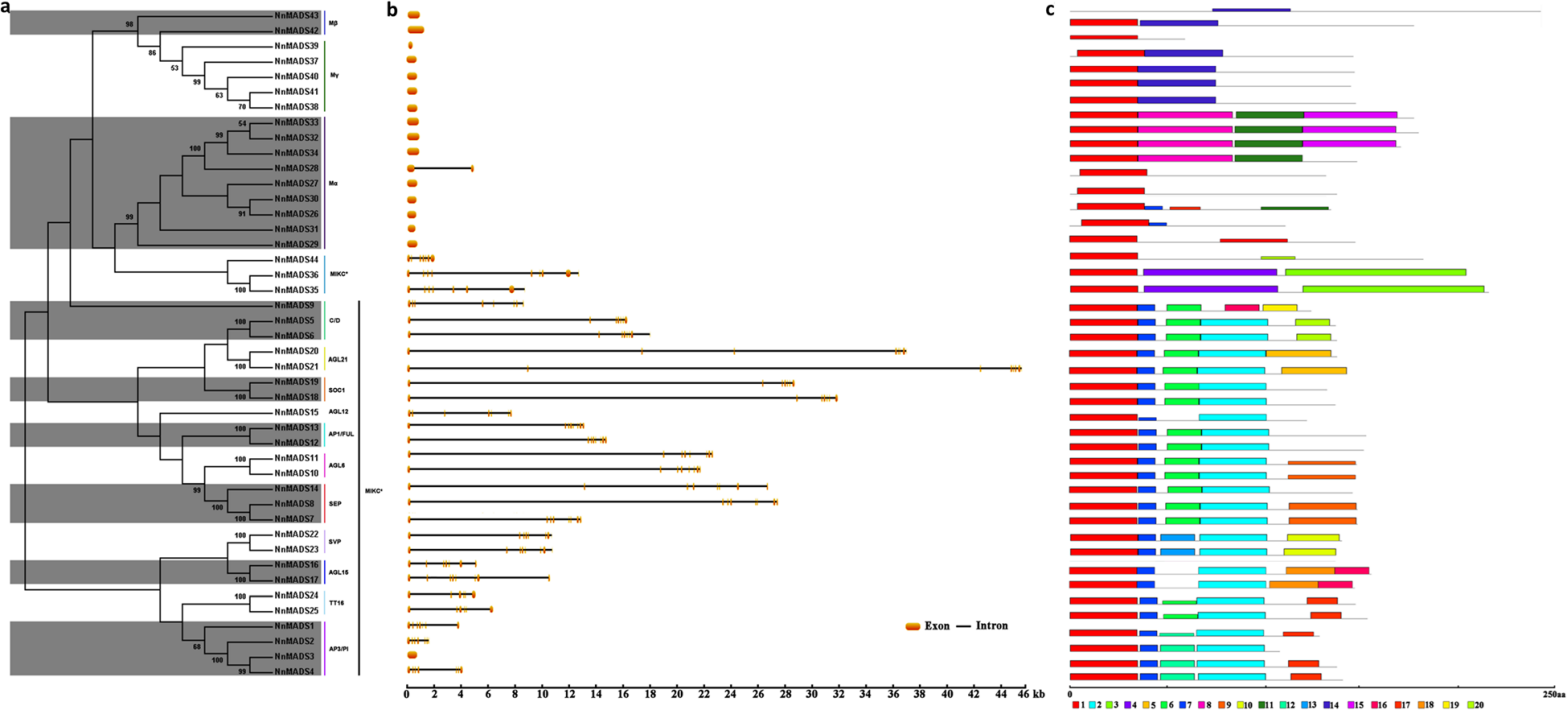
Phylogenetic relationship, gene structure, and motif analyses of *NnMADS* genes. **a** A neighbor-joining tree was constructed based on the alignment of full-length amino acid sequences of NnMADS protein. **b** The gene structures of each *NnMADS* gene without the untranslated regions (UTRs). Analysis was carried out using the Gene Structure Display Server tool (http://gsds.cbi.pku.edu.cn/). The size scale is indicated at the bottom. **c** Putative motifs in NnMADS proteins were analyzed by MEME. The boxes with different colors represent different putative motifs, and are marked by the numbers at the bottom. The scale bar represents the length of proteins in terms of amino acids

To investigate the gene structure of MADS-box genes in lotus, the full lengths of cDNA and genomic DNA sequences of 44 *NnMADS* genes obtained from NCBI database were used for phylogenetic analysis alone, which is consistent with that along with the data from *Arabidopsis* and rice (Fig. 1b; Fig. 2a). The structures of these *MADS* genes could also show the phylogenetic relationship among them (Fig. 2b). Except for *NnMADS3* with only one exon, all the other MIKC-type (Type II) genes had an exon-intron structure with more than six exons (Fig. 2b). In contrast, there was almost no intron for Type I MADS-box genes, except for *NnMADS28* that contained one intron (Fig. 2b).

A total of twenty conserved motifs among the 44 lotus MADS-box proteins were identified with MEME motif search tool (Fig. 2c). Motif 1 exists in 43 members except for NnMADS43, which contained only one motif (Fig. 2c). Motif 2 and 7 were present in all MIKC^c^ MADS-box proteins except for NnMADS9 which lacked motif 2 (Fig. 2c). The E class (NnMADS7/8) and AGL6 homologous (NnMADS10/11) subfamily contained motif 9, whereas motif 10 and 13 were conserved in SVP subgroup (NnMADS22 and NnMADS23). Motif 16 was considered as the typical B class motif (Fig. 2c). Motif 12 and motif 17 represented individual pattern of preservation among NnMADS1/2/3/4 in clade B class subfamily. In addition, motif 17 was also found in NnMADS24/25, members of the TT16 clade. AG cluster members including NnMADS5/6 contained a special motif 20. Other MADS-box proteins had some specific motifs in the subfamily, though the motifs were inconsistent. Different members of Mα type MADS-box genes contained different motif numbers, such as NnMADS26, NnMADS28, and NnMADS32/33/34 contained more than 3 motifs, whereas NnMADS27 and NnMADS29/30/31 had less than two motifs. Mβ and Mγ groups had two motifs at most. NnMADS39 and NnMADS43 contained one unspecific motif (Fig. 2c).

**Fig. 3:**
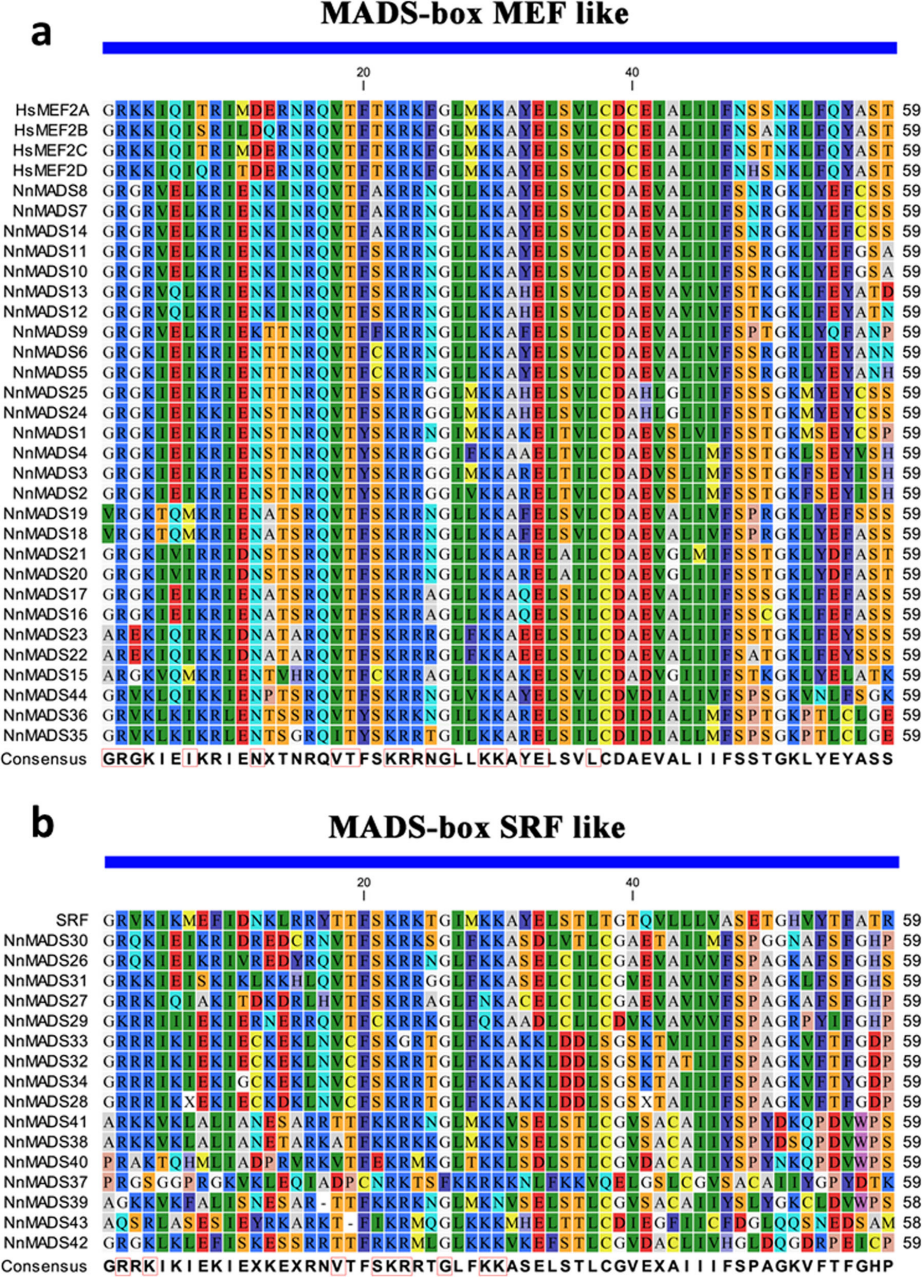
Conservative analysis on MADS-box domain among different NnMADS proteins. **a** MADS-box MEF like alignment. **b** MADS-box SRF like alignment. HsMEF2A, HsMEF2B, HsMEF2C, HsMEF2D and SRF sequences were retrieved from *Homo sapiens* database as reference. Protein sequences correspond to the conserved regions on all NnMADS. In the consensus line, uppercase letters represent identity in more than 50% of sequences and X represents less than 50% identity. The red box marked letters indicate the specific residues involved in a conserved feature (referred to MEF and SRF domain from NCBI)

The conserved core M region of NnMADS proteins had ~ 59 amino acids, which is consistent with those in other species [20]. There are two types of M region named as type I (SRF-like) and type II (MEF-like), respectively. To assess the conservation of M region among the NnMADS proteins, members of type I and type II were subjected to alignment analysis along with the representatives of each type from *Homo sapiens*, which showed that both types are highly conserved with type II (MEF-like) being more conserved (Fig. 3).

### Spatial expression of *NnMADS*s in floral organs and subcellular localization of several members of NnMADSs

**Fig. 4:**
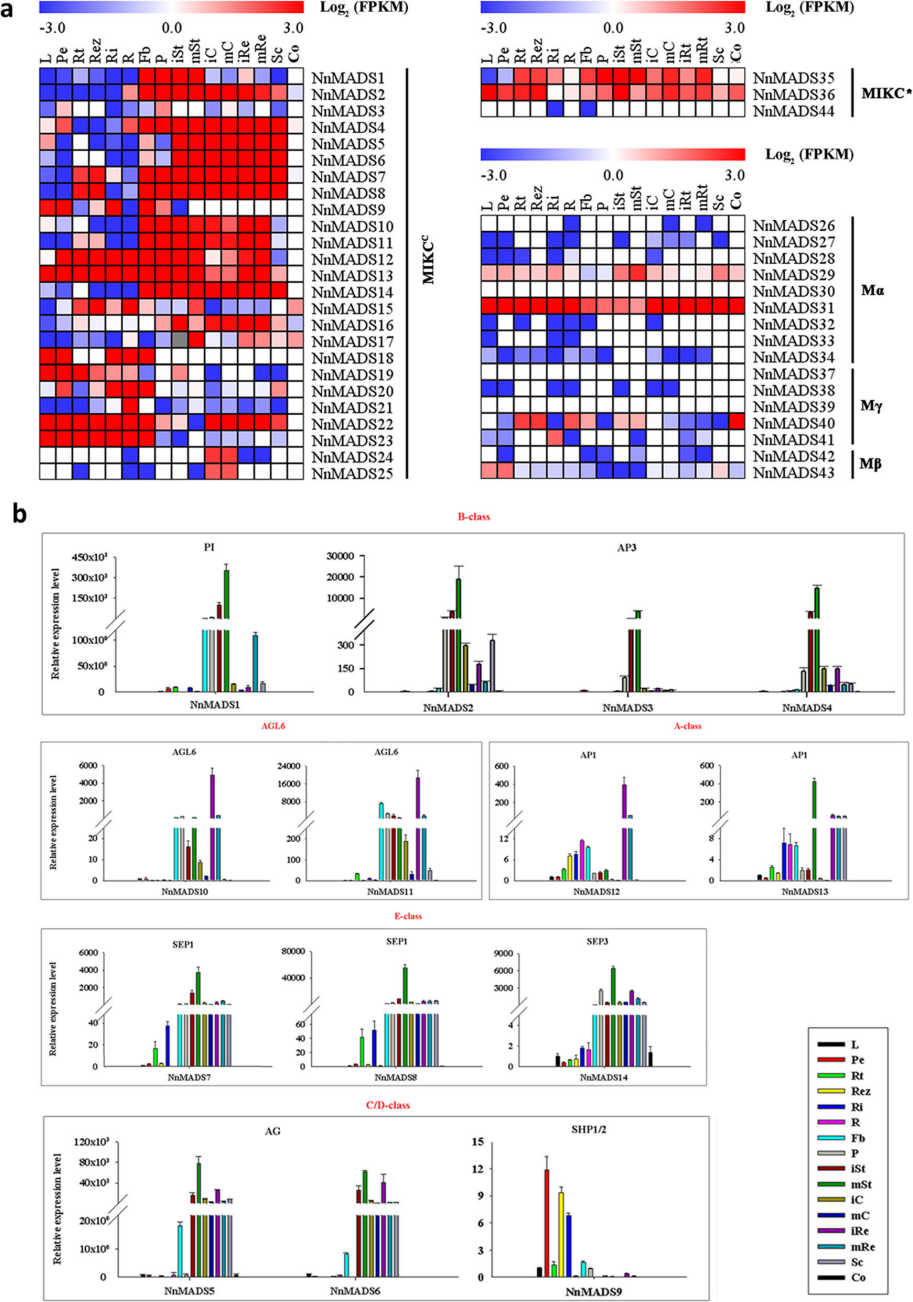
The expressional patterns of *NnMADS* genes in different tissues of *N. nucifera*. **a** The left panel shows the MIKC^c^ sub-type members of type II MADS box genes; the upper panel of right side shows MIKC* sub-type members of type II MADS box genes, and the lower panel of the right side shows those of the type I. The FPKM values were collected from the online data as mentioned in M&M, and used for drawing the heatmap. **b** qRT-PCR analysis on the relative expressions of 14 representative *NnMADS* genes in different tissues of *N. nucifera*. Transcript level of each *NnMADS* were first normalized to those of the housekeeping gene *NnActin* and then compared to the expression level of each gene in leaf. Data are means ±SD (*n* = 3). L: Leaf; Pe: Petiole; three parts of the rhizome, such as Rt: rhizome tip, Rez: Rhizome elongation zone, Ri: Rhizome internode; R: Root; Fb: Flower bud; P: Petal; Being collected stamen, carpel, receptacle before pollination named as immature stamen (iSt), immature carpel (iC), immature receptacle (iRe); Being collected stamen, carpel, receptacle after pollination named as mature stamen (mSt), mature carpel (mC), mature receptacle (mRe); Sc: Seed coat; Co: Cotyledon

The expression of a given gene might be related to its function. To explore the expressional patterns of *NnMADSs*, the transcriptome data of 16 tissues including leaf, petiole, rhizome (including tip, elongation zone, and internode), root, flower bud, petal, stamen (immature and mature), carpel (immature and mature), receptacle (immature and mature), and seed (seed coat and cotyledon) from a previous study were used for further analysis [53]. The data for these 44 *NnMADSs* were extracted and used to draw a heat-map to show their expression styles (Fig. 4a, Table S4). The type I *NnMADS* genes generally had low or undetectable expression in all the tissues, except for *NnMADS29*, *NnMADS30*, *NnMADS40*, *NnMADS41*, and *NnMADS43* (Fig. 4a). Both *NnMADS29*, *NnMADS30* belong to Mα subtype and are ubiquitously expressed among different tissues (Fig. 4a). *NnMADS40* showed preferential expression in root, rhizome, and cotyledon, *NnMADS41* was rhizome internode-specific and *NnMADS43* was leaf and petiole specific (Fig. 4a). As for type II *NnMADS* genes, MIKC^*^ subtype contained two ubiquitously expressed members and one with no detectable expression (Fig. 4a), whereas the MIKC^c^ subtype could be categorized into three expressional patterns named as floral organ preferential, vegetative tissue preferential, and ubiquitous patterns (Fig. 4a). The floral organ preferential expressed genes included *NnMADS1*, *NnMADS2*, *NnMADS4–8*, *NnMADS10*, *NnMADS11*, *NnMADS16*, and *NnMADS17*; vegetative tissue preferential expressed genes included *NnMADS9*, *NnMADS15*, *NnMADS18*, *NnMADS19*, *NnMADS20*, and *NnMADS23*; and the ubiquitous expressed genes included *NnMADS12*, *NnMADS13*, and *NnMADS22*. Specifically, *NnMADS24* and *NnMADS25* were expressed only in carpel (Fig. 4a). Since we were more concerned with floral organogenesis, qRT-PCR was conducted to confirm the accumulation of those MIKC^c^ subtype *MADS* genes (*NnMADS1–14*) with high expression in the floral organs (Fig. 4b, Table S5). A Comparison between qRT-PCR and RNA-Seq data showed that they were generally consistent with each other (Fig. S2).

**Fig. 5:**
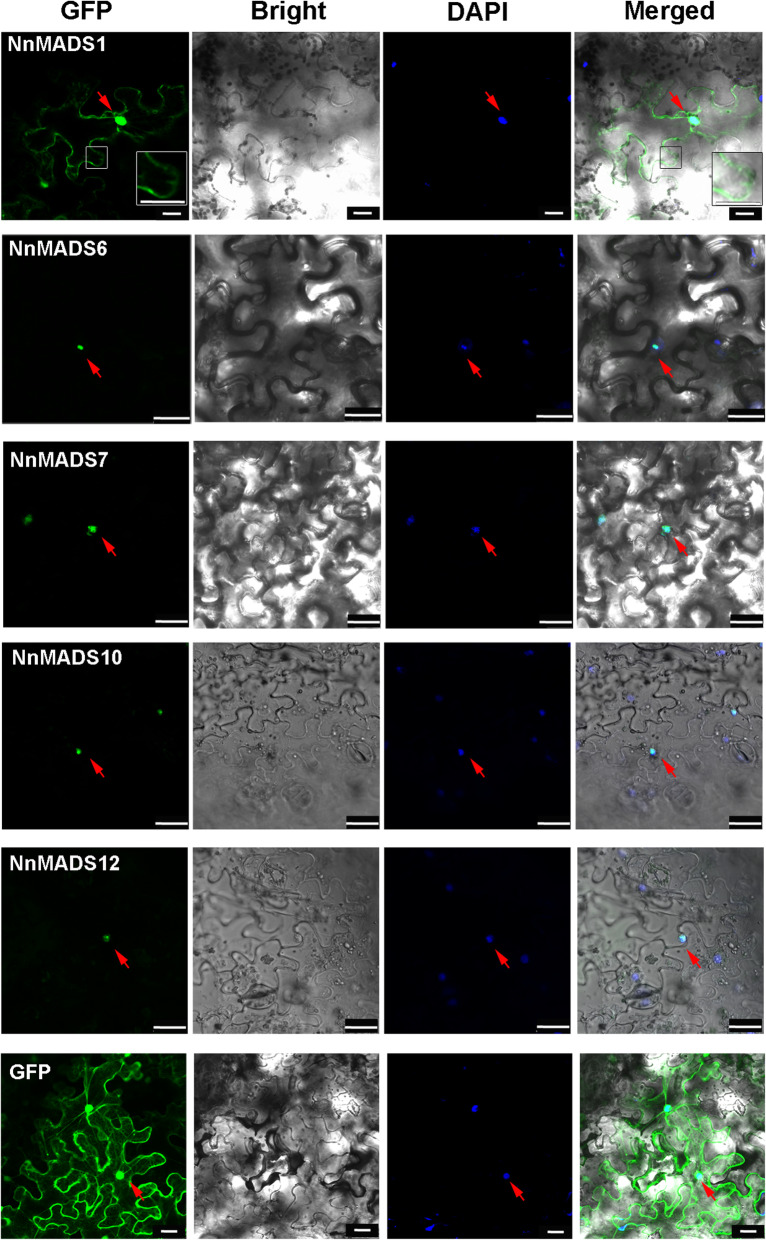
Subcellular localization of six representative NnMADSs fused with green fluorescent protein. NnMADSs were constructed into plasmid with fusions of GFP and driven by the CaMV35S promoter. DAPI was used to mark nuclei. Panels from left to right refer to GFP, Bright, DAPI, and Merged images, respectively. Protein fusions co-localized with DAPI markers are marked with red arrows. The boxes are enlarged images for cytomembranes. Scale bars = 25 μm

Since correct localization of proteins is very important for their functions, the subcellular localization of NnMADS proteins was also investigated through transient transformation of tobacco leaves. We chose several representative MADS-box family members including A class gene (*NnMADS12*), B class gene (*NnMADS1*), C class gene (*NnMADS*6), E Class genes (*NnMADS7*, *NnMADS14*), and *AGL6* gene (*NnMADS10*), and fused them with green fluorescent protein (GFP) for the subcellular localization analysis. All of them were localized in the nuclear (Fig. 5). Interestingly, the GFP signals of *NnMADS1* were also localized in the cytomembrane (Fig. 5).

### Overexpression of *NnMADS14* in *Arabidopsis*

**Fig. 6:**
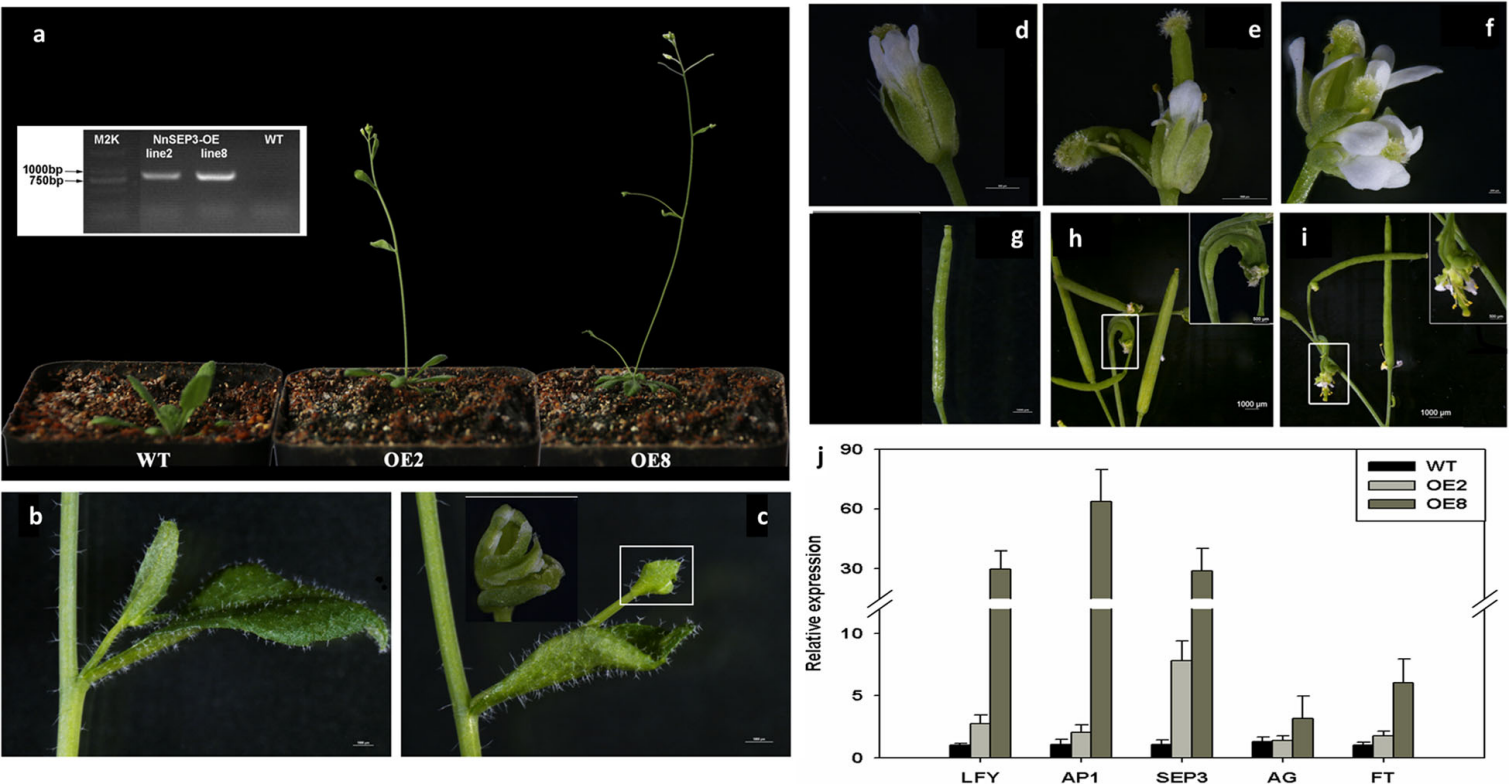
Phenotyping of 35S:*NnSEP3* transgenic Arabidopsis. **a** Early flowering of two transgenic Arabidopsis lines. The inserted panel shows the overexpression of *NnSEP3* in the two lines (OE2 and OE8). **b** Cauline leaf of wide type Arabidopsis. **c** Transition of cauline leaf to flower in 35S:*NnSEP3* transgenic Arabidopsis. The inserted panel shows the transitional flower. **d** Normal flower in wide type Arabidopsis. **e** Flower with two pistils in the OE2 line. **f** Transition of single flower into double flowers with two pistils in the OE8 line. **g** Normal silique in wide type Arabidopsis. (**h**, **i**) Aberrant silique of OE2 (**h**) and OE8 (**i**) lines. The inserted panels show the enlarged images of the area in the white rectangles. Bars are 500 μm. **j** Expression analysis of the endogenous flowering and leaf development-related genes in *Arabidopsis*. WT: wide type, OE: 35S::*NnSEP3*. *LFY*, *AP1*, *SEP3*, *AG*, and *FT* were analyzed by qRT-PCR in leaves

It is known that E-class genes are necessary for the genesis of each floral organ, including petal, stamen, and pistil [14]. All the floral organs display a sepal-like phenotype in *Arabidopsis* E-class genes triple mutant *spe1spe2spe3*. And in tetra-mutant of *spe1spe2spe3spe4*, all the floral organs were transited into leaf-like organs [16]. In lotus, there are three E-class MADS-box genes which include *NnMADS7*, *NnMADS8*, and *NnMADS14*. According to their expressional patterns, *NnMADS14* was the only one showing floral organ specificity because the former two both had high expression in root and rhizome (Fig. 4). Therefore, we selected *NnMADS14* as the candidate gene to verify its function in Arabidopsis. The *NnMADS14* driven by 35S promoter construct was transformed into *Arabidopsis* (Col-0). A total of 10 transgenic lines were obtained, among which two of the T3 lines showed an early flowering phenotype (Fig. 6a). The expression of *NnMADS14* gene was then checked in these two lines, which was confirmed to be overexpressed (Fig. 6a). Besides early flowering, the overexpression lines also showed phenotypes of transition from leaf to floral organ (Fig. 6b, c) and formation of double pistils or double flowers (Fig. 6d-i).

To explore the underlying mechanisms of these phenotypes, we then checked the expression of several key genes, including *AtLFY*, *AtAP1*, *AtAG*, and *AtFT*, that regulate the flowering time and inflorescence formation in the two *Arabidopsis NnMADS14* overexpression lines. The qRT-PCR results showed that the expression of both *AtLFY* and *AtAP1* genes were dramatically increased along with the overexpression of *NnMADS14*, whereas *AtAG* and *AtFT* had only a tiny increase in their expression (Fig. 6j).

### Expression of *NnMADS14* in different lotus strains

**Fig. 7:**
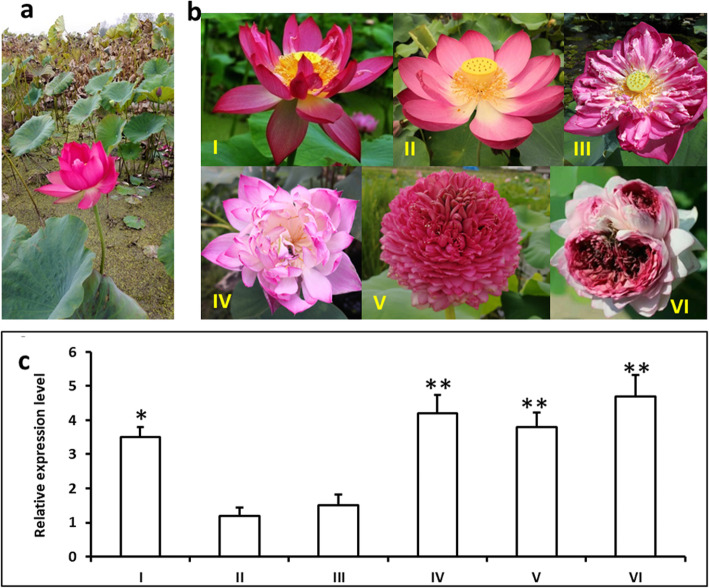
Expression analysis of *NnSEP3* gene in the flower bud of different lotus strains. **a** Flower of the longer flowering lotus in late autumn. The flowering time expand from June to November. **b** Flowers of different lotus species. **c** Expression levels of *NnSEP3* gene among different strains. I, longer flowering lotus; II, ordinary lotus; III, strain with stamen petaloid; IV, strain with ovary petaloid; V, all-double-petalled-flower strain; VI, strain with multi- all-double-petalled-flowers in one flower bud

Based on the phenotype of *Arabidopsis NnMADS14* overexpression lines, it seems that the expression level of this gene is positively related to the flowering and floral organogenesis. To confirm if it is also true in lotus, six different lotus strains were selected to analyze the expression of *NnMADS14* gene. These strains displayed different flowering time and flower shapes (Fig. 7a, b). qRT-PCR results showed that *NnMADS14* gene was expressed in the flower buds of all the strains (Fig. 7c). However, the expression levels were higher in the strains showing either longer flowering time or pistil petaloid (Fig. 7c).

## Discussion

Because of its importance in plant floral organ and fruit development, MADS-box gene family has gained increasing attention from the scientific community. To-date, *MADS-box* genes have been systematically characterized in more than 34 different plant species [11, 22, 39–42]. Through searching against the released genome database of *N. nucifera* ‘China Antique’ [43], a total of 44 MADS-box genes were determined, whereas only 40 MADS-box genes were annotated in the genome of another wild strain of sacred lotus [44]. This calls for further improvement of lotus genome data, which might lead to the discovery of more *NnMADSs*. Genes of this family could be sorted into two types with five sub-types. Phylogenetic analysis of these *NnMADS* genes along with those from *Arabidopsis* and rice showed that the divergence of different types and sub-types of this gene family occurred before the evolution of these three different plant species. This divergence might have occurred during the genesis of embryophyte or even before (Fig. 1b). Generally, the basal species contain less *NnMADS* genes than those highly evolved species [54], which seems to be determined by the whole genome duplication (WGD). During evolution, at least two WGD events occurred in *Arabidopsis* and rice [55, 56], whereas lotus experienced only one WGD with the absence of the γ WGD [43, 44]. Ka/Ks ratio approximated to one showed that no selection mutation occurred. These suggested that *N. nucifera* could be considered as a basal plant model for studying the evolution of MADS-box function.

Different with type II genes containing abundant transcript variants, all type I genes belong to monophyletic lineages, which is consistent with the hypothesis that the birth- and death-evolution rate of type I genes is higher than that of type II in angiosperms [57]. Most of the type I genes have a simple structure with only one exon (Fig. 2), which is similar to that in rice, grape, and *Arabidopsis* [22, 39, 42]. In contrast, structures of type II MADS-box genes are more complicated, with several of them containing more than ten exons (Fig. 2). The protein structures of type II are also more complicated than those of type I. Together, the type II MADS-box genes might be subjected to more intricate regulation. Along with previous reports, it also indicated that type II genes might be functionally more important than type I genes for plants, although some of type I genes were shown to play important regulatory roles in plant reproduction as well [58, 59].

Among the type II genes, MIKC^c^ sub-type is best known for its plant specific and importance for floral organogenesis. The phylogenetic analysis showed that genes of this sub-type could be further divided into 12 clades (Fig. 1b), with the FLC clade being absent in both lotus and rice. It is known that the *FLC* genes regulate the flowering time through controlling vernalization and vernalization-independent pathways [60]. It seems that the FLC clade is absent in plant species, including rice, cotton, and orchid, that do not require vernalization for flowering [39, 41, 61]. This suggests that lotus does not require vernalization for flowering.

At least 14 *NnMADSs* belong to the well-known classic ‘ABCDE’ model genes in plant [5–11], which consists of class A (AP1/FUL), class B (AP3/PI), class C/D (AG/STK/SHP1/2), and class E (SEP1/2/3/4) [9, 13, 16]. Most of the ‘ABCDE’ model genes displayed a floral organ preferential expression pattern (Fig. 4), indicating their potential roles in the floral organogenesis of lotus. Subcellular localization of representative members of A, B, C/D, and E class genes verified their nuclear localization (Fig. 5). Because the E class genes are important for the development of each floral organ based on the ‘ABCDE’ model, one of its homolog in lotus *NnMADS14* was selected for functional characterization in *Arabidopsis*, which proved its importance in floral organogenesis (Fig. 6). The result suggests that *NnMADS14* functions mainly through regulating the expression of *LFY* and *AP1* genes. Although there is still no stable transformation system for lotus, the expression of *NnMADS14* gene among different lotus germplasm verified that this gene is highly related to the flowering time and floral organogenesis in lotus (Fig. 7). Similar functions of *SEP*-like genes in floral organ formation were also proved in many other plants, such as *Phalaenopsis* orchid, *Prunus mume,* and soybean [62–64]. In strawberry (that develops from the receptacle of the flower) SEP1/2 homolog FaMADS9 plays an important role in receptacle development and regulates ripening programs [23, 65].

## Conclusions

In conclusion, a genome-wide search identified a total of 44 MADS-box genes in the lotus genome. Phylogenetic analysis showed the potential divergence time of MADS-box gene family, and the lack of *FLC* clade in plant species that do not require vernalization. Systematic analyses on their expressional patterns and subcellular localization showed some potential candidates *NnMADS* genes involved in floral organogenesis. Among them, the function of a homolog of *SEPs* gene *NnMADS14* was verified in *Arabidopsis* system, along with its expression in different lotus germplasm showing obvious difference in flowering time and flower shape. The comprehensive information on lotus MADS-box gene family might help to further understand the mechanism underlying its floral organ development, and hence contribute to the breeding of high value ornamental lotus.

## Methods

### Plant materials, RNA extraction, and cDNA synthesis

*Nelumbo nucifera* cultivar ‘China Antique’ (named by Prof. Guozheng Huang) was grown in experimental pools in Wuhan, China (30°32′45″N114°24′52″E) by the authors, which is identical with the sequenced one [43], and has been cultivated for several decades in Wuhan Botanical Garden, Chinese Academy of Sciences. The 16 tissues included leaf, petiole, rhizome (including tip, elongation zone, and internode), root, flower bud, petal, stamen (immature and mature), carpel (immature and mature), receptacle (immature and mature), and seed (seed coat and cotyledon) were collected before 10:00 am in July. For sample harvesting, it is unnecessary to obtain any permission from any authority. All the samples were frozen with liquid nitrogen immediately after harvesting, and then kept in − 80°C freezer until used for RNA extraction.

The RNA reagent (OminiPlant RNA Kit, CWBIO, China) was used to extract the total RNAs. During extraction, genomic DNA was removed with RNase-free DNase I (Thermo, Shanghai, China). The RNAs were reversed with HiScript II One Step RT-PCR Kit (Vazyme, China) to synthesize complementary DNAs (cDNAs) synthesis according to instructions of the Kit.

### Database search and identification of MADS-box family genes in lotus

MADS-box protein sequences of *Arabidopsis* and rice were retrieved from TAIR (http://www.arabidopsis.org/) and Rice Genome Annotation Project (http://rice.plantbiology.msu.edu/), respectively. *N*.*nucifera* MADS-box protein sequences were obtained from the lotus database (http://lotus-db.wbgcas.cn/) and were blasted in NCBI to get the full-length CDS and genomic sequence. To confirm and identify the putative *N. nucifera* MADS-box genes, SRF-TF domain (PF00319) retrieved from Pfam 31.0 (http://pfam.xfam.org/) was searched against the hidden Markov model (HMM, https://www.ebi.ac.uk/Tools/hmmer/search/phmmer). The detailed information is shown in Table S1. MADS-box sequences from other species used in this study were obtained from previous researches [40, 41, 52]. The evolutionary relationships among these species were constructed using the taxonomy tool in NCBI (https://www.ncbi.nlm.nih.gov/Taxonomy/CommonTree/wwwcmt.cgi) and the phylip tree format was downloaded. Then, the phylip tree was generated by phylip software version 3.695 (http://evolution.genetics.washington.edu/phylip/getme-new1.html).

### Construction of physical map and phylogenetic analysis

The open accessed software Mapchart (v2.3.2) was used to analyze distribution of *MADS-box* genes in the lotus genome, specifically in the top ten biggest megascaffolds [66]. Multiple sequences were aligned by MUSCLE using default parameters [67]. The phylogenetic trees were constructed by the neighbor-joining (NJ) algorithm using MEGA7.026 [68] with the Jones-taylor-thomton (Jtt) model. The bootstrap method was applied in phylogeny test with 1000 replications. The gaps and missing data treatment were set using the pair-wise deletion option to certify the dissimilatory regions that could generate the topology of NJ tree.

### Analysis of *MADS-box* genes’ structure and duplication

The lotus MADS-box family protein sequences were analyzed using the MEME software version 5.0.1 (http://meme-suite.org/doc/cite.html) [69]. The parameters were set as follows: repetitions could be any number, maximum number of motifs = 20, 6 ≤ width ≤ 200. SMART tool (http://smart.embl-heidelberg.de/) was used to confirm the MEME motifs. ClustalX software and CLC Sequence Viewer 8.0.0 software (https://www.qiagenbioinformatics.com/products/ clc-sequence-viewer-direct-download/) were used to conduct the multiple sequence alignments, and generated the image. Using the Gene Structure Display Server tool (http://gsds.cbi.pku.edu.cn), the exon/intron gene structure was carried out with mapping the CDSs to genomic sequences (without UTR). Potential gene duplications with major standard analysis were conducted as follows: (1) length of sequence alignment covers longer gene more than 75%, and (2) identity of the alignment being more than 75% [70]. The values of *Ka* and *Ks* were performed by TBtools (https://github.com/CJ-Chen/TBtools) [71].

### Expression analysis using *N. nucifera* RNA-seq data

For the expression profile of *N. nucifera* MADS-box genes, RNA-seq data was downloaded from NCBI SRA (PRJNA492157, PRJNA503979, and PRJNA428028) and lotus database (http://lotus-db.wbgcas.cn/), and then used for analysis. Sixteen tissues, including leaf, petiole, tip, elongation zone, internode, root, flower bud, petal, immature anther, mature-anther, immature carpel, mature carpel, immature receptacle, mature receptacle, seed coat, and cotyledon were presented by values of FPKM (fragments per kilobase of exon model per million mapped reads) (Fig. S3, Table S4). Mev software version 4.9.0 was used to generate the heat-map of expression of *NnMADS* genes.

### Analysis of gene expression by quantitative real-time PCR

Homologous classic ABC(D) E model genes were picked out from the MADS-box family in lotus as candidate genes. Fourteen MADS-box genes were chosen to quantify the transcription levels in different tissues. Actin was used as an internal control. The primer sets are listed in Table S6. The qRT-PCR reactions were performed using the SYBR Green Master Mix (BioRad, http://www.bio-rad.com/) as previously described [51]. Three biological replicates, each with three technical repeats, were analyzed. And the relative gene expression was calculated by 2^−△△Ct^ comparative threshold cycle (Ct) method [72]. The data were indicated as mean ± SD.

### Transgenic and subcellular localization analysis

The candidate MADS-box family members coding domain sequence (CDS) were amplified from *N. nucifera* cDNA via PCR using high-fidelity thermostable DNA polymerase. The primers for these genes were designed with Primer Premier 5.0. The PCR products were cloned into pMD®18-T vector (TaKaRa). For sequencing, the PCR products were cloned into a vector, and then transformed into DH5α *E.coli* cells. For Nicotiana transformation, they were cloned into pMDC83 vector fused with GFP and driven by CaMV 35S promoter. The primers are listed in Table S6. The recombinant plasmids were isolated and used to transform tobacco leaf according to the protocol described by Sparkes et al. [73]. After infiltration, the plant was allowed to grow for 2 or 3 days, the transformed leaves were observed on a confocal microscope (Leica). The DAPI Staining Solution (Beyotime) was used to stain the nucleus.

For transgenic analysis, the construct 35S::*NnSEP3*-GFP was transformed into *Arabidopsis thaliana* (Col-0) using *Agrobacterium*-mediated floral dip method [74]. *Arabidopsis thaliana* plants were cultivated in a growth chamber maintained at 22 °C and a 16 h/8 h (light/dark) photoperiod. The T3 homozygous transgenic lines were used for further study.

## Supplementary Information

**Additional file 1: Table S1.** MADS-box genes identified in *N. nucifera*. **Table S2.** Segmental and tandem duplications of paralogous MADS-box pairs in lotus. **Table S3.** Categorization of MADS-box genes family in three plant genomes. **Table S4.** FPKM value of *NnMADSs*. **Table S5.** The relative expressions of 14 representative *NnMADS* genes under qRT-PCR analysis. **Table S6.** The primers of *NnMADS* genes in lotus.

**Additional file 2: Figure S1.** Distribution of MADS-box genes in lotus genome. **a** Mapping of the *NnMADS* genes in megascaffold-1 ~ − 10 of lotus genome. The unit of the length is Mb. **b** The number of *NnMADS* genes in each megascaffold. c The density of MADS-box genes in megascaffold − 1 ~ − 10. The unit is Mb/gene. **Figure S2.** The correlation analysis of RNA-seq and qRT-PCR data. **a** The correlation of fourteen MADS-box genes in lotus tissues. **b** The correlation of NnMADS12 and NnMADS13 after removing two off-line data. The low expression or not detected data were not considered. **Figure S3.** The different tissues of *N. nucifera*. L: Leaf; four parts of rhizome, such as Pe: Petiole, Rt: rhizome tip, Rez: Rhizome elongation zone, Ri: Rhizome internode; R: Root; Fb: Flower bud; P: Petal; St: Stamen; C: carpel; iRe: immature Receptacle; mRe: mature Receptacle; Sc: Seed coat; Co: Cotyledon.

## Data Availability

All data generated or analyzed during this study are included in this published article and its supplementary information files.

## Abbreviations

AG: Agamous
AP: Apetala
CDS: Coding Domain Sequence
DEF: Deficiens
Ct: Threshold Cycle
FLC: Flowering Locus
FPKM: Fragments per Kilobase of Exon Model per Million Mapped Reads
FUL: Fruitful
MADS: MCM1, AG, DEF and SRF
MAF1/FLM: Mads affecting flowering
MCM1: Minichromosome maitenance
MEF: Myocyte enhacer factor
*NnMADS*: *Nelumbo nucifera* MADS
PI: Pisittala
qRT-PCR: Quantitative Real-Time PCR
SEP: Sepallata
SHP: Shatterproof
SOC: Suppressor of overexpression of constans
SRF: Serum response factor
STK: Seedstick
VP: Short vegetative phase
TT16: Transparent testa16
WGD: Whole genome duplication
