# Supplementary material for: Genome-wide identification of MADS-box gene family in sacred lotus (Nelumbo nucifera) identifies a SEPALLATA homolog gene involved in floral development
Source: BMC Plant Biol. 2020 Oct 29;20:497. doi: 10.1186/s12870-020-02712-w (PMC7599106; doi:10.1186/s12870-020-02712-w)
Supplement: Supplementary file 2 — Additional file 2: Figure S1. Distribution of MADS-box genes in lotus genome. a Mapping of the NnMADS genes in megascaffold-1 ~ − 10 of lotus genome. The unit of the length is Mb. b The number of NnMADS genes in each megascaffold. c The density of MADS-box genes in megascaffold − 1 ~ − 10. The unit is Mb/gene. Figure S2. The correlation analysis of RNA-seq and qRT-PCR data. a The correlation of fourteen MADS-box genes in lotus tissues. b The correlation of NnMADS12 and NnMADS13 after removing two off-line data. The low expression or not detected data were not considered. Figure S3. The different tissues of N. nucifera. L: Leaf; four parts of rhizome, such as Pe: Petiole, Rt: rhizome tip, Rez: Rhizome elongation zone, Ri: Rhizome internode; R: Root; Fb: Flower bud; P: Petal; St: Stamen; C: carpel; iRe: immature Receptacle; mRe: mature Receptacle; Sc: Seed coat; Co: Cotyledon. [file 12870_2020_2712_MOESM2_ESM.pdf]

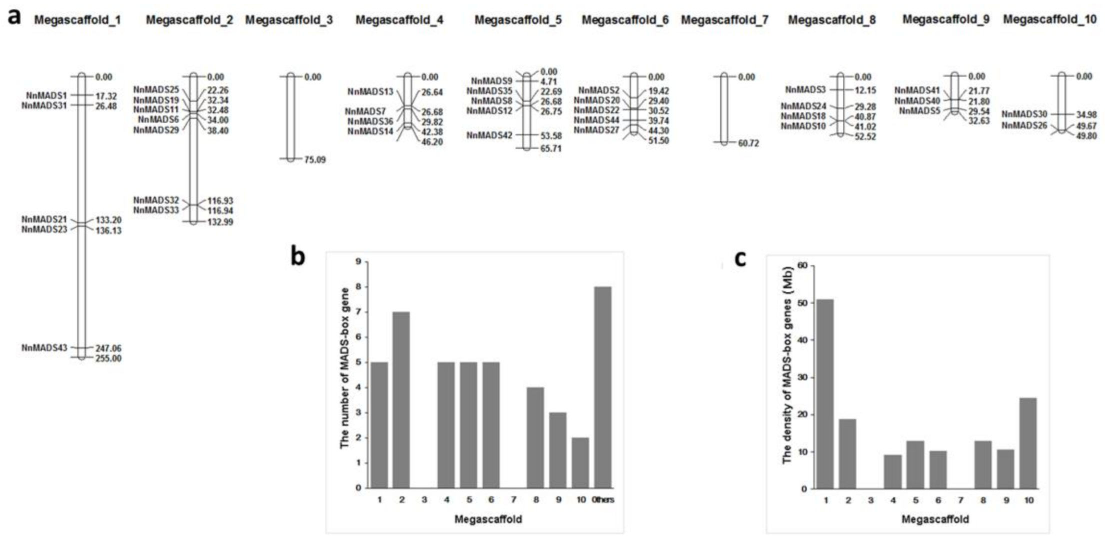

Figure S1. Distribution of MADS-box genes in lotus genome. a Mapping of the *NnMADS* genes in megascaffold-1~-10 of lotus genome. The unit of the length is Mb. **b** The number of *NnMADS* genes in each megascaffold. c The density of MADS-box genes in megascaffold -1~-10. The unit is Mb/gene.

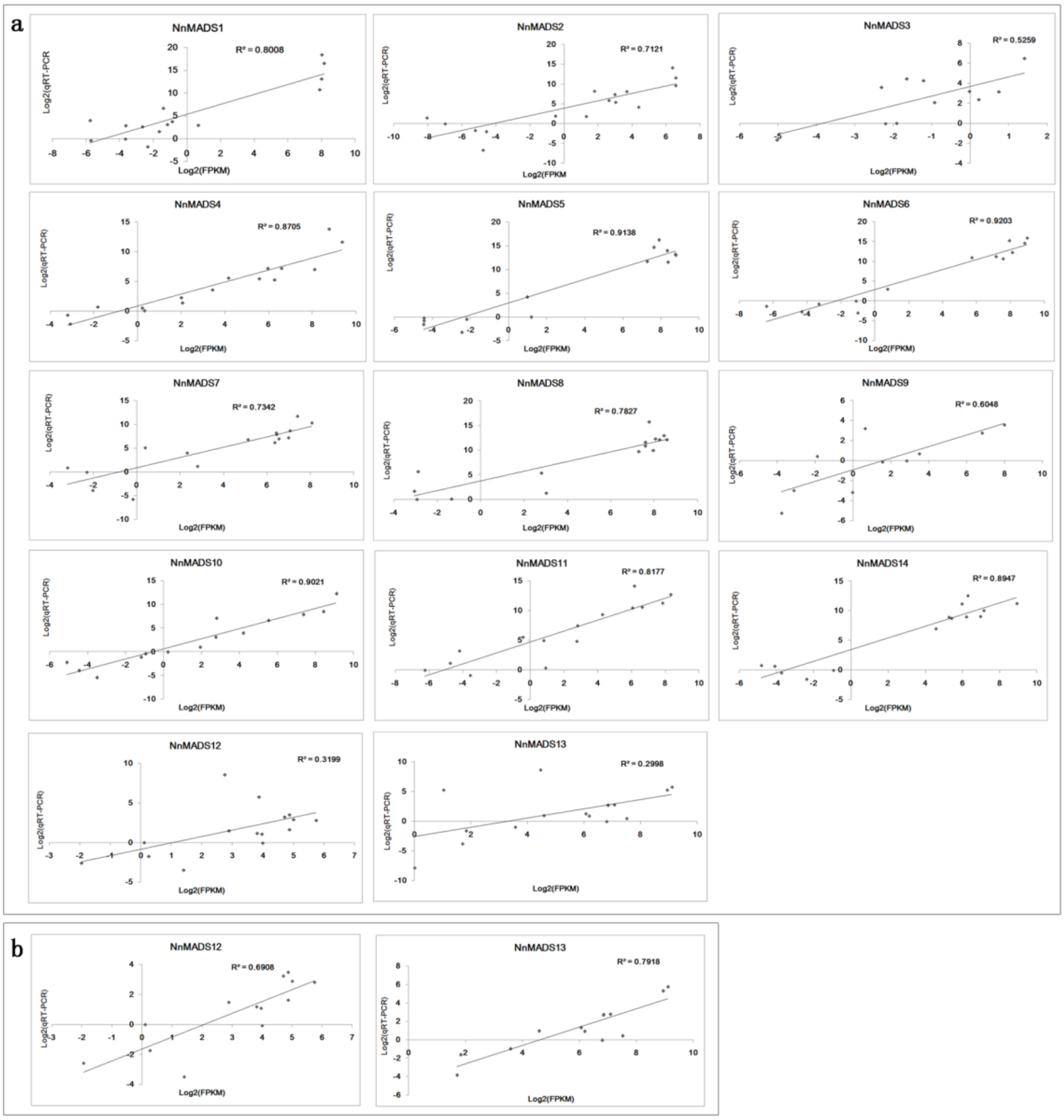

Figure S2. The correlation analysis of RNA-seq and qRT-PCR data. a The correlation of fourteen MADS-box genes in lotus tissues. **b** The correlation of NnMADS12 and NnMADS13 after removing two off-line data. The low expression or not detected data were not considered.

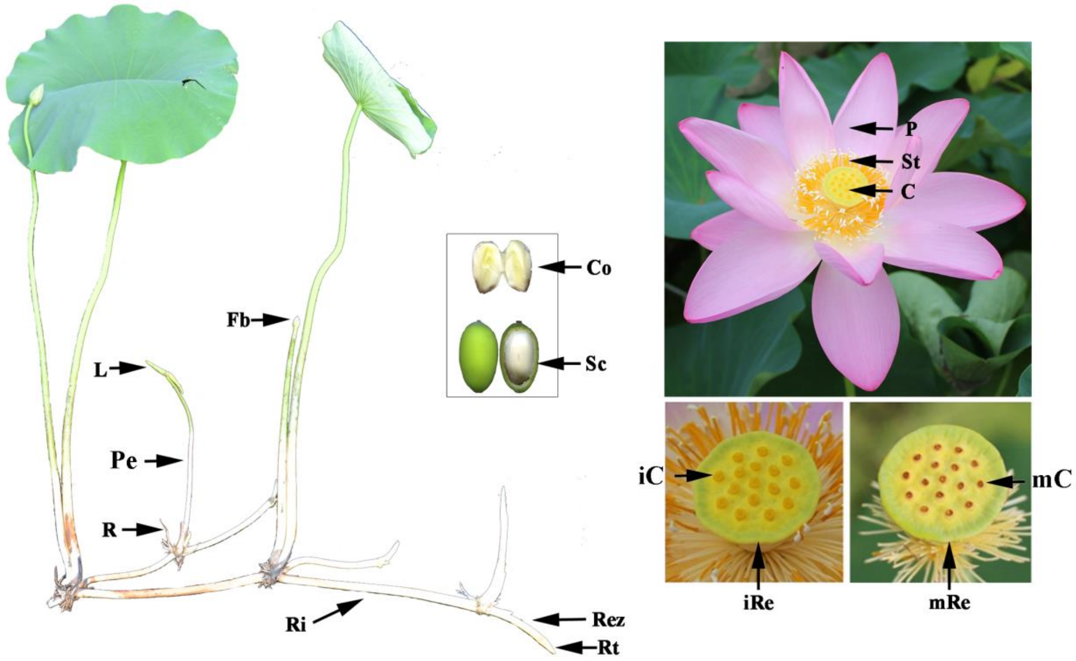

Figure S3. The different tissuses of *N. nucifera*. L: Leaf; four parts of rhizome, such as Pe: Petiole, Rt: rhizome tip, Rez: Rhizome elongation zone, Ri: Rhizome internode; R: Root; Fb: Flower bud; P: Petal; St: Stamen; C: carpel; iRe: immature Receptacle; mRe: mature Receptacle; Sc: Seed coat; Co: Cotyledon.
